# Wound management, healing, and early prosthetic rehabilitation: Part 2 - A scoping review of physical biomarkers

**DOI:** 10.33137/cpoj.v7i2.43716

**Published:** 2024-12-05

**Authors:** H Williams-Reid, A Johannesson, A Buis

**Affiliations:** 1 Department of Biomedical Engineering, Faculty of Engineering, University of Strathclyde, Glasgow, Scotland.; 2 Össur Clinics EMEA, Stockholm, Sweden.

**Keywords:** Amputation, Scoping Review, Wound Healing, Surgical Site Healing, Physical Biomarkers, Physical Markers of Healing, Residuum Healing, Residual Limb Healing, Wound Management, Early Prosthetic Rehabilitation

## Abstract

**BACKGROUND:**

The timely provision of load-bearing prostheses significantly reduces healthcare costs and lowers post-amputation mortality risk. However, current methods for assessing residuum health remain subjective, underscoring the need for standardized, evidence-based approaches incorporating physical biomarkers to evaluate residual limb healing and determine readiness for prosthetic rehabilitation.

**OBJECTIVE(S):**

This review aimed to identify predictive, diagnostic, and indicative physical biomarkers of healing of the tissues and structures found in the residual limbs of adults with amputation.

**METHODOLOGY:**

A scoping review was conducted following Joanna Briggs Institute (JBI) and PRISMA-ScR guidance. Searches using “biomarkers”, “wound healing”, and “amputation” were performed on May 6, 2023, on Web of Science, Ovid MEDLINE, Ovid Embase, Scopus, Cochrane, PubMed, and CINAHL databases. Inclusion criteria were: 1) References to physical biomarkers and healing; 2) Residuum tissue healing; 3) Clear methodology with ethical approval; 4) Published from 2017 onwards. Articles were assessed for quality (QualSyst tool) and evidence level (JBI system), and categorized by study, wound, and model type. Physical biomarkers that were repeated not just within categories, but across more than one of the study categories were reported on.

**FINDINGS:**

The search strategy identified 3,306 sources, 157 of which met the inclusion criteria. Histology was the most frequently repeated physical biomarker used in 64 sources, offering crucial diagnostic insights into cellular healing processes. Additional repeated indicative and predictive physical biomarkers, including ankle-brachial index, oxygenation measures, perfusion, and blood pulse and pressure measurements, were reported in 25, 19, 13, and 12 sources, respectively, providing valuable data on tissue oxygenation and vascular health.

**CONCLUSION:**

Ultimately, adopting a multifaceted approach that integrates a diverse array of physical biomarkers (accounting for physiological factors and comorbidities known to influence healing) may substantially enhance our understanding of the healing process and inform the development of effective rehabilitation strategies for individuals undergoing amputation.

## INTRODUCTION

### 1: OVERALL RATIONALE, AIMS, AND OBJECTIVES

Wound healing is the biological process of tissue repair following damage,^1^ such as amputation surgery or prosthetic-use induced deep tissue injuries (DTIs). The process comprises four interrelated stages: hemostasis, inflammation, proliferation, and tissue remodeling.^2–4^ It demands a high degree of cellular coordination, introducing several avenues through which impairments can occur. Consequently, wound healing can be stalled (also referred to as non-healing, impaired, or chronic) not by one isolated factor, but by several smaller contributing issues.^5^ Common post-amputation surgical site healing complications include infection, pain, hematomas, tissue necrosis, poor residual limb formation, recurrent ulceration, wound dehiscence, and stitch abscesses.^6,7^ Persistent complications, in other words, poor healing, can necessitate revision surgeries or even re-amputation at more proximal levels.^6^

Despite the complexity of wound healing, current healing assessments remain largely surface-level and subjective. This is especially relevant for major lower limb amputees, who typically receive a customized prosthetic limb within 3 to 20 weeks post-surgery, depending on wound healing.^8,9^ Prosthetic fitting significantly improves mobility, physical health, and quality of life,^9–11^ yet determining residual limb readiness remains subjective and inconsistent.^12^

Clinical judgment, based on superficial wound assessments, varies widely, and there are no standardized guidelines for evaluating readiness.^12–14^ Factors such as wound healing, pain management, and limb volume are considered, but specific measurable indicators are lacking. Recent studies highlight debates around key clinical decisions, such as whether to use rigid or soft dressings in the immediate post-operative stage to promote healing.^15,16^ Moreover, individuals awaiting amputation frequently present with multiple comorbidities that complicate the healing process. A leading cause of amputation is diabetes-related complications,^17^ yet hyperglycemia can lead to vascular stiffening, microvascular dysfunction, reduced tissue oxygenation, and, consequently, impaired wound healing.^18^

This variability in clinical practices underscores the need for more objective measures, such as biomarkers, to assess wound healing and readiness for prosthetic use. Biomarkers, defined by the U.S. FDA (Food & Drug Administration) as measurable indicators of biological processes or responses to treatment,^19^ offer a way to reduce the subjectivity inherent in current practices. However, there is limited research on using biomarkers to monitor healing and support early prosthetic rehabilitation post-amputation. Existing studies, like those investigating tissue composition changes during prosthetic use,^20^ focus on mature residual limbs, while early-stage limbs face higher risks of issues like ulceration and volume fluctuation, complicating socket fit.^21^ Exploring these early stages is crucial for successful prosthetic rehabilitation and preventing further surgeries. To meet this research need, a scoping review was developed and implemented with the following aim:

Identify predictive, diagnostic, and/or indicative biomarkers (physical, chemical, or other) of healing of the tissues and structures found in the residual limbs of adults with amputation.

To meet this aim, the following objectives were compiled:

**1)** Collate and synthesize the reported definitions of healing and non-healing in the literature investigating healing of the tissues and structures found in the residual limbs of adults with amputation.
**2)** Identify and collate physical biomarkers predictive, diagnostic, and/or indicative of healing repeated in sources investigating healing of the tissues and structures found in the residual limbs of adults with amputation.
**3)** Identify and collate chemical biomarkers predictive, diagnostic, and/or indicative of healing repeated in sources investigating healing of the tissues and structures found in the residual limbs of adults with amputation.
**4)** Assess the quality and levels of evidence of sources investigating healing of the tissues and structures found in the residual limbs of adults with amputation.

The term “physical” refers to biomarkers like wound pH, temperature, or collagen levels detected through histochemical staining,^22^ while “chemical” pertains to markers present in wound tissue, fluids, serum/blood, sebum, saliva, or sweat, such as cytokines or matrix metalloproteinases. Indicative biomarkers suggest the presence of a condition or physiological state but are not definitive. Predictive biomarkers provide prognostic information, indicating the likelihood of developing a condition or predicting a patient's response to treatment. Diagnostic biomarkers confirm the presence of a specific disease or condition, or in this context, definitively identify the progression of healing.

### 2: PART 2 - RATIONALE, AIMS, AND OBJECTIVES

This article (Part 2) addresses objective 2 and constitutes the second instalment in a series of three articles, each of which sequentially examines objectives 1 to 3. As concluded in Part 1,^23^ there exists a significant lack of consensus and standardization in defining healing and non-healing within the literature that investigates the healing of the tissues and structures found in the residual limbs of adults with amputations. Most approaches fail to consider deeper tissue healing and the mechanical properties of the tissue essential for functionality, particularly in the context of prosthetic use.^23^ To address this, Part 1 outlined steps for developing a tailored and relevant scale that incorporates biomarkers for assessing wound healing in the context of residual limbs post-amputation.

Physical biomarkers assess the macro-level physiological properties of a biological system, such as heart rate, which indicates cardiac functionality. These biomarkers are typically measured in real-time or continuously, offering the potential for ongoing monitoring of wound healing. For instance, recent work by Patel et al.^24^ synthesized research on wearable electronics for skin wound monitoring and healing, noting the development of sensors capable of real-time monitoring of physical biomarkers, including pH, temperature, moisture, and oxygen. Day et al.^12^ similarly concluded that future research should assess transcutaneous oxygen perfusion, along with other non-invasive measures of blood flow and perfusion, as a more objective means of tracking the progression of healing over time. Notably, transcutaneous oxygen pressure (TcPO_2_) was the only objective measure employed among the 15 sources reviewed in their study.^12^ Previous research has indicated that a TcPO_2_ value below 40 mmHg is associated with a 24% increased risk of healing complications in lower limb amputations compared to values above 40 mmHg.^25^

Physical biomarkers are already widely utilized in various healthcare settings for different applications. For example, peripheral oxygen saturation (SpO_2_) has been employed by the UK National Health Service (NHS) to detect early deterioration in patients with COVID-19 in primary and community care settings.^26^ Medically certified pulse oximetry fingertip devices were distributed to patients, enabling the rapid real-time measurement of oxygen saturation levels without the need for blood samples.^26^ Furthermore, SpO_2_ has also been shown to correlate with wound healing; Park et al.^27^ demonstrated that, during the early stages of wound healing, oxygen saturation can drop to a maximum of 85%, indicating a hypoxic wound environment. As healing progresses, oxygen saturation typically increases and is maintained within the normal range of 95% to 100% by the end of the healing process, as observed in a rat cutaneous wound model.^27^ These existing pulse oximetry systems demonstrate significant potential for adaptation and reapplication in the monitoring of residual limb healing and early prosthetic rehabilitation. This serves as a clear example of how the requirement to identify and develop techniques for quantifying biomarkers within the proposed healing assessment scale can be effectively addressed.

In conclusion, physical biomarkers represent promising objective measures for inclusion in the development of an assessment scale of residual limb healing post-amputation. Therefore, the aim of this review was to:

Identify predictive, diagnostic, and/or indicative physical biomarkers of healing in the tissues and structures found in the residual limbs of adults with amputations.

To achieve this aim, the following objectives have been established:

**1)** Identify and compile physical biomarkers that are predictive, diagnostic, and/or indicative of healing as reported in sources investigating the tissues and structures of residual limbs in adults with amputations.
**2)** Identify and summarize the techniques used to quantify these physical biomarkers in studies focused on the healing of tissues and structures in residual limbs of adults with amputations.
**3)** Assess the quality and levels of evidence in sources investigating the healing of tissues and structures found in the residual limbs of adults with amputations.

## METHODOLOGY

Given the novelty of the research question and the broad array of sources available on biomarkers, a scoping review was deemed the most appropriate approach to address the research question. The complete review methodology has been previously detailed in Part 1.^23^ In brief, the review adhered to the Preferred Reporting Items for Systematic Reviews extension for Scoping Reviews (PRISMA-ScR) checklist and guidance^28,29^ and followed the Joanna Briggs Institute (JBI) guidelines.^30–33^ Data management was conducted using Excel Version 2303 (Microsoft, Washington, USA) operating on Windows 11 Version 22H2 (Microsoft, Washington, USA).

### 1: INCLUSION CRITERIA AND SEARCH STRATEGY

The first screening phase, focusing on titles and abstracts, applied primary inclusion criteria including references to biomarkers of wound healing, healing of tissues found in the residual limb, and publications from 2017 onwards. Due to the limited research specifically addressing biomarkers for residual limb healing, the inclusion criteria were expanded to encompass literature on biomarkers of healing, requiring that participants have a clearly defined wound in tissues and structures comparable to those of an amputation residuum. In the second phase of full-text screening, additional criteria were introduced, including clear and reproducible methodologies, ethical approval (where applicable), and the involvement of human participants (aged 18+) or murine models. To ensure a comprehensive review, sources were considered from diverse contexts, such as home, hospital community, and academic institutions, and across multiple disciplines, including healthcare professionals and engineers. Additionally, to mitigate bias towards high-income countries and Western publication bias,^34,35^ studies from any geographical region were included, provided they were available in the English language due to the primary reviewer's language limitations.

An exhaustive list of terms derived from the research question was generated and the search strategy was piloted. Finalized search terms, based on terms “biomarker”, “amputation”, and “wound healing”, were then applied to several databases, including Web of Science, MEDLINE (hosted on the Ovid platform), Embase (hosted on the Ovid platform), Scopus, Cochrane, PubMed, and CINHAHL. The extensive number of sources generated during the initial searches prompted a reassessment of the inclusion criteria. Additionally, the rapid advancements in wound healing biomarkers^36^ underscored the necessity for more recent data. A recent scoping review examined prognostic factors (biomarkers) associated with ulcer healing, a common diabetic complication that can precede amputation,^37^ specifically focused on sources published before 2017.^38^ In light of this context, it was decided to include only sources published in or after 2017, thereby ensuring the relevance and timeliness of the reviewed literature. Search results were exported and managed in EndNote 20 (Version 20.2.1, Clarivate, 2021), where duplicates were removed.

### 2: DATA EXTRACTION, ANALYSIS, AND PRESENTATION

Data extraction (including study type and characteristics, and physical biomarkers) was performed by the primary reviewer using a pre-defined tool for sources that passed both screening rounds. The QualSyst too^l39^ (chosen for its quantitative and reproducible quality assessment) and the JBI levels of evidence^40^ were used to evaluate study quality and evidence levels respectively. A prevalence of poor-quality or low-level evidence would indicate the need for methodological improvements in biomarker research. All extracted data, including references for included sources, are openly accessible in the review's dataset.^41^

Due to the nature of a scoping review, a meta-analysis is not considered appropriate.^30^ Instead, basic descriptive analyses, such as frequency counts of key concepts, were prioritized. Extracted biomarkers were subject to frequency counts, and evidence levels and quality scores were compiled. The included sources are categorized based on study type (randomized controlled trial, case study, observational study, or bench research), wound type (diabetic, amputation, or other), and model type (human, murine, or other, such as cell lines). Each category provides distinct insights into wound healing, contributing to a comprehensive understanding from multiple perspectives.

Physical biomarkers that were observed repeatedly, not only within categories but also across multiple study categories, are visually represented in a tree-map graph and are further analyzed in the discussion through comparison with existing literature. This manuscript focuses on these recurring biomarkers, based on the assumption that repetition indicates a stronger evidence base for the biomarker's use, thus supporting further research on these biomarkers. A separate descriptive section summarizes the methodologies for biomarker quantification.

## RESULTS

### 1: OVERALL RESULTS

#### 1.1: Search Strategy Results

As detailed in Part 1,^23^ the search strategy implemented in May 2023 resulted in the identification of 7,041 sources. Following the removal of 3,735 duplicate records, a total of 3,306 titles and abstracts were screened (see Part 1 for the PRIMSA diagram^23^). Ultimately, 219 articles were selected for data extraction. Exclusions were based on factors such as review articles study type, unclear methodologies, and lack of ethical approval. Of the 219 articles selected, 157 reported on physical biomarkers, and were therefore the focus of this Part 2 review.

#### 1.2: Quality and Levels of Evidence

For a detailed reporting and discussion of the quality and levels of evidence of all 219 sources that meet the inclusion criteria for the overall review aim, please refer to Part 1.^23^ The levels of evidence across the 157 included sources were variable encompassing both the highest and lowest tiers of evidence. For instance, within the Effectiveness category, only 1 study^42^ (of 157 included sources) was graded as 1.b, and 6 studies^43–48^ received a grade of 1.c; however, a significant majority, 79 sources^49–127^ were rated at 5.c (the lowest level of evidence).

All studies evaluated were quantitative, with none receiving a limited quality score. Specifically, 79% of all studies were demonstrated strong quality,^43,44,46,47,49–52,54,55,57–63,67–71,74,76–81,91–99,–101–108,111,115,117,118,121–187^ 19% were rated as good quality,^42,45,53,56,64–66,72,73,89,90,100,109,110,112–114,116,119,188–197^ and only 4% were classified as adequate quality.^48,75,120,198^

#### 1.3: Study Types and Characteristics

Of the 157 included sources, 79 were classified as bench research studies (**Table 1**- Study Categories 9 to 13), while only 3 were identified as case-controlled studies.^149,151,162^ This data was further analyzed based on wound type and model type (**Table 1**). Notably, bench research studies focusing on diabetic wounds using mouse models constituted the largest study category, comprising 36 sources.

**Table 1:** Overview of the study types of all 157 included sources utilizing physical biomarkers. The table categorizes the included sources by study type, wound type, and model type and provides the reference number for the category used throughout the review. The number of included sources and percentage of the 157 included sources in each category are detailed.

Study Type			Category Reference Number	Number (%) of Included Sources	Included Source References
Randomised Controlled Trial			1	7 (4%)	42, 135, 156, 172, 193, 194, 196
Case-Controlled Study			2	3 (2%)	149, 151, 162
Observational	Prospective	Diabetic Wounds	3	14 (9%)	45, 54, 138, 140, 141, 143, 153, 173, 176, 178, 182, 188, 191, 192
		Amputation	4	5 (3%)	44, 137, 139, 179, 198
		Other Wounds	5	9 (6%)	46–48, 144, 146, 152, 161, 164, 181
	Retrospective	Diabetic Wounds	6	13 (8%)	43, 70, 113, 130, 133, 134, 147, 148, 160, 163, 165, 168, 175
		Amputation	7	14 (9%)	128, 132, 142, 145, 166, 169, 177, 180, 183–185, 187, 189, 195
		Other Wounds	8	13 (8%)	136, 150, 154, 155, 157–159, 167, 170, 171, 174, 186, 190
Bench Research	Diabetic Wounds	Rat Models	9	22 (14%)	50, 59, 68, 71, 80, 81, 83, 86, 89, 91, 94, 97, 98, 100, 106, 108, 111, 112, 114, 116, 119, 123
		Mouse Models	10	36 (23%)	49, 52, 53, 57, 60, 61, 63–65, 69, 72, 73, 77–79, 82, 85, 87, 88, 90, 93, 95, 102, 103, 109, 110, 115, 117, 121, 122, 124–126, 129, 131, 197
		Other Models	11	5 (3%)	58, 62, 74, 75, 96
	Other Wounds	Rat/Mouse Models	12	13 (8%)	51, 55, 56, 66, 67, 76, 84, 92, 99, 101, 104, 105, 118
		Other Models	13	3 (2%)	107, 120, 127

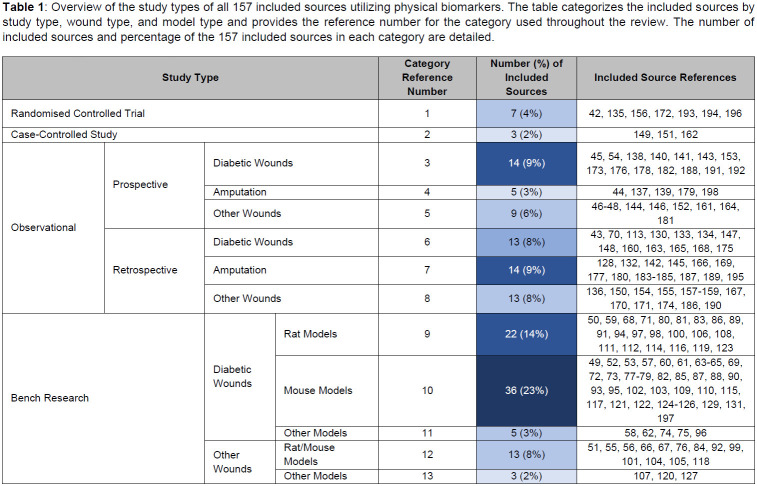

In Categories 1 to 8 (**Table 1**), human participants were employed, with sample sizes ranging from a minimum of 2 (a case-controlled study^151^) to 7,187 (an observational retrospective study^145^). Within the human participant studies that provided gender information (71 of 78 sources) sample genders ranged from a minimum of 20% male^161^ to 99% male^145^ (**Table 2**). Medians of the mean ages were all above 60 years, with means ranging from 27.1^146^ years to 77.3 years.^195^ In some sources, age was instead described by ranges and median ages (**Table 2**). 34 (44%) of the 78 human participant studies investigated diabetic wounds, 21 (27%) focused on amputations (some of which were a result of a diabetic wound), and 23 (29%) investigated other wounds (**Table 2**). Examples of other wounds included acute lower extremity wounds,^196^ anterior cruciate ligament tear reconstruction,^199^ chronic foot ulcers,^47,190^ and appendectomy surgical sites.^167^

**Table 2:** The characteristics of the included sources involving human participants, specifically wound type, sample size, sample gender, and sample age, are detailed for Study Categories 1 to 8 (refer to **Table 1**). The notation “No. (%) of references” indicates the number and percentage of sources that provide characteristic information relative to the total number of sources within that category (T.G. = treatment groups; C.G. = control groups; No. = number).

	Study Category							
	1	2	3	4	5	6	7	8
**Wound Type Totals**								
Diabetic	**5** (42, 135, 172, 193, 194)	**2** (149, 151)	**14** (45, 54, 138, 140, 141, 143, 153, 173, 176, 178, 182, 188, 191, 192)	0	0	**13** (43, 70, 113, 130, 133, 134, 147, 148, 160, 163, 165, 168, 175)	0	0
Amputation	**1** (156)	**1** (162)	0	**5** (44, 137, 139, 179, 198)	0	0	**14** (128, 132, 142, 145, 166, 169, 177, 180, 183–185, 187, 189, 195)	0
Other	**1** (196)	0	0	0	**9** (46–48, 144, 146, 152, 161, 164, 181)	0	0	**13** (136, 150, 154, 155, 157–159, 167, 170, 171, 174, 186, 190)
**Sample Size Totals**								
Range (Min-Max)	16–50	2–58	10–684	10–556	5–735	92–1032	13–7187	45–637
Median	33	20	66	19	60	232	121	120
No. (%) of References	**7** (100%)	**3** (100%)	**14** (100%)	**5** (100%)	**9** (100%)	**13** (100%)	**14** (100%)	**13** (100%)
**Sample Gender (% Male) Totals**								
Range (Min-Max)	40%–82%	47%–100%	35%–84%	60%–73%	20%–90%	45%–83%	29%–99%	54%–78%
Median	61%	50%	67%	64%	63%	62%	71%	66%
No. (%) of References	**5** (71%) (42, 135, 156, 193, 196)	**3** (100%)	**13** (93%) (45, 54, 138, 140, 141, 143, 153, 176, 178, 182, 188, 191, 192)	**5** (100%)	**8** (89%) (46, 47, 144, 146, 152, 161, 164, 181)	**11** (85%) (43, 70, 113, 130, 133, 134, 160, 163, 165, 168, 175)	**14** (100%)	**12** (92%) (136, 150, 154, 155, 157–159, 167, 170, 171, 174, 186)
**Sample Mean Age (Years) Totals**								
Range (Min-Max)	T.G.: 55.0–69.0; C.G.: 52.1–64.7	60.2–61.5	48.0–67.0	49.0–74.0	27.1–72.6	54.5–72.5	61.5–77.3	56.0–74.9
Median	T.G: 64.2; C.G.: 62.0	60.9	61.2	68.4	65	61.2	66.5	72
No. (%) of References	**6** (86%) (42, 135, 156, 172, 193, 196)	**2** (67%) (151, 162)	**13** (93%) (45, 54, 138, 140, 141, 143, 153, 173, 176, 178, 182, 188, 192)	**4** (80%) (44, 137, 139, 198)	**6** (67%) (46, 47, 144, 146, 152, 181)	**12** (92%) (43, 70, 113, 130, 134, 147, 148, 160, 163, 165, 168, 175)	**12** (86%) (128, 132, 142, 145, 166, 177, 180, 183, 184, 187, 189, 195)	**9** (69%) (136, 155, 157–159, 170, 171, 174, 186)
**Sample Age Range (Years) Totals**								
Range (Min-Max)	NA	45–65	20–89	23–66	28–81	17–96	26–96	NA
No. (%) of References	NA	**1** (33%) (149)	**6** (43%) (45, 143, 173, 176, 182, 191)	**2** (40%) (44, 179)	**3** (33%) (161, 164, 181)	**5** (38%) (147, 148, 163, 165, 175)	**4** (29%) (177, 180, 187, 189)	NA
**Sample Median Age (Years) Totals**								
Range (Min-Max)	NA	NA	NA	NA	NA	72.5	47.0–62.0	31.0–71.2
Median	NA	NA	NA	NA	NA	72.5	54.5	68.4
No. (%) of References	NA	NA	NA	NA	NA	**1** (8%) (133)	**2** (14%) (169, 185)	**3** (23%) (150, 154, 167)

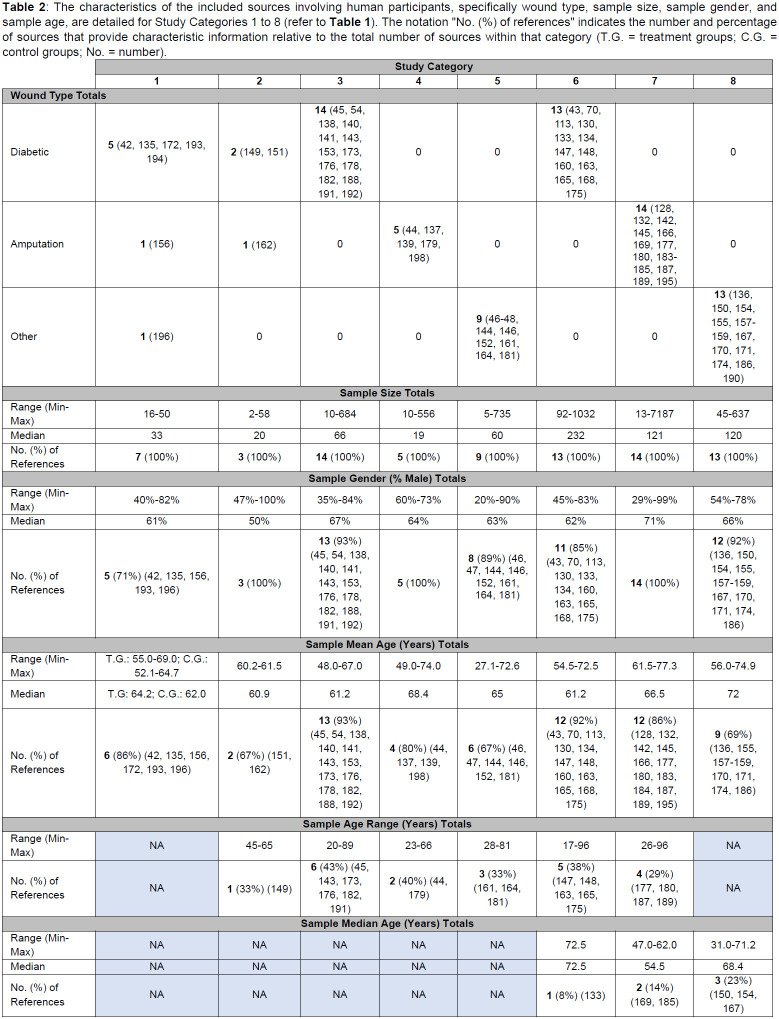

The synthesis of the 79 bench research studies (Study Categories 9 to 13) revealed complex sample characteristics. Among the 71 studies employing rat or mouse models, 46 (65%) used exclusively male rodents, 7 (10%) used only females, and the remainder either did not specify gender or used both. Seven of the eight studies in “other models” (Categories 11 and 13) utilized cell lines (animal and human), wound healing assays (scratch assays), and/or human tissue samples.^58,74,75,96,107,120,127^ The remaining study employed a mathematical model.^62^ Of the 79 bench research studies, 63 (80%) focused on diabetic wounds (**Table 1**- Study Categories 9 to 11), with only one study^92^ examining hind limb amputation in Sprague-Dawley rats. The remaining 15 studies investigated other wounds, including sciatic nerve injuries (cut and crush injuries; 2 sources^67,84^), traumatic injuries (musculoskeletal trauma and blast-associated injuries; 3 sources^55,104,105^), skin wounds (7 sources^51,56,66,76,99,101,118^), and general wound cell models (includes wound/scratch assays; 3 sources^107,120,127^).

### 2: REPEATED PHYSICAL BIOMARKERS

**Figure 1:**
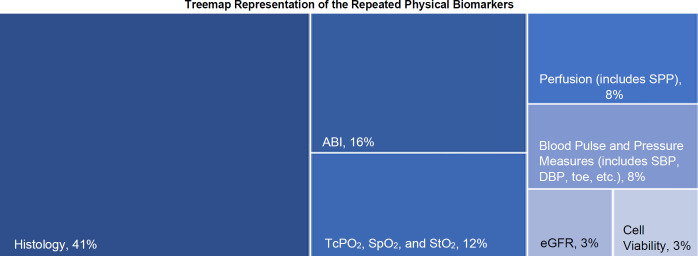
Treemap visualization displaying the frequencies of the repeated physical biomarkers. A biomarker was considered “repeated” if it was used in more than one source within a study category and appeared in more than one study category. The occurrence of these biomarkers in the 157 included sources is presented as a percentage (ABI = ankle-brachial index; TcPO_2_ = transcutaneous oxygen pressure; SpO_2_ = saturation of peripheral oxygen; StO_2_ = skeletal muscle oxygen saturation; SPP = skin perfusion pressure; SBP = systolic blood pressure; DBP = diastolic blood pressure; eGFR = estimated glomerular filtration rate).

The most frequently reported physical biomarker was histology, which encompasses measures such as collagen deposition and the degree of angiogenesis, all determined through microscopic analysis of sectioned and stained tissue samples. Histology was employed in 64 sources representing 41% of the 157 included sources (**Table 3** and **Figure 1**). Additional physical biomarkers, utilized not only within but also across various source types, included ankle-brachial index (ABI), oxygenation measures (such as TcPO_2_ [transcutaneous partial oxygen pressure], SpO_2_ [peripheral oxygen saturation], and StO_2_ [tissue oxygen saturation]), perfusion, and blood pressure and pulse measurements. These biomarkers were reported in 25 (11%), 19 (9%), 13 (6%), and 12 (5%) sources, respectively (**Table 3**).

**Table 3:** A comprehensive breakdown of the repeated physical biomarkers. A biomarker was considered “repeated” if it was used in more than one source within a study category and appeared in more than one study category. The occurrence of these biomarkers in the 157 included sources is presented, along with their representation across the various study categories (see **Table 1**; ABI = ankle-brachial index; TcPO_2_ = transcutaneous oxygen pressure; SpO_2_ = saturation of peripheral oxygen; StO_2_ = skeletal muscle oxygen saturation; SPP = skin perfusion pressure; SBP = systolic blood pressure; DBP = diastolic blood pressure; eGFR = estimated glomerular filtration rate).

Repeated Physical Biomarkers	Sources			Study Categories		
	Frequency	% of Included Sources	References	Frequency	% of Categories	Categories Included
Histology	64	41%	50–53, 56, 57, 59–61, 63–68, 71–73, 76–83, 85–91, 93, 95, 97–103, 106, 108–112, 114–119, 121–126, 131, 172, 194, 197	4	31%	1, 9, 10, 12
ABI	25	16%	113, 128, 132, 141, 143, 145, 154, 155, 157, 161, 163–166, 168, 173, 174, 176, 181, 183, 186, 188, 192, 193, 196	6	46%	1, 3, 5, 6, 7, 8
TcPO_2_, SpO_2_, and StO_2_	19	12%	45, 48, 54, 70, 113, 132, 139, 141, 144, 147, 148, 153, 165, 168, 179–181, 188, 191	5	38%	3, 4, 5, 6, 7
Perfusion (includes SPP)	13	8%	42, 46, 48, 142, 155–157, 161, 164, 170, 174, 189, 196	4	31%	1, 5, 7, 8
Blood Pulse and Pressure Measures (includes SBP, DBP, Toe Pressure, etc.)	12	8%	113, 134, 147, 148, 160, 165, 175, 177, 187, 188, 192, 195	3	23%	3, 6, 7
eGFR	5	3%	43, 133, 138, 168, 173	2	15%	3, 6
Cell Viability	5	3%	74, 96, 107, 120, 127	2	15%	11, 13

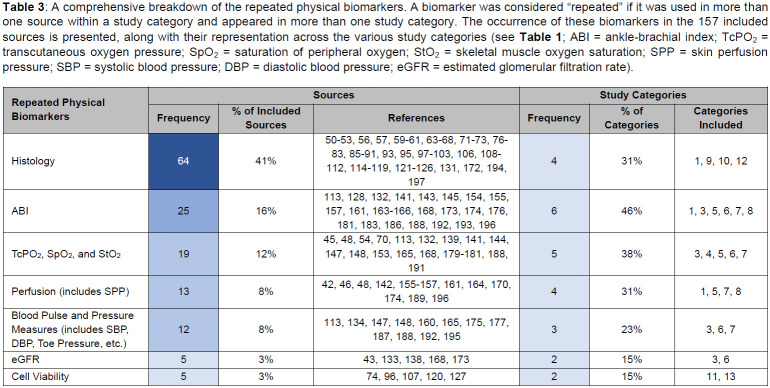

### 3: MEASUREMENT TECHNIQUES OF REPEATED PHYSICAL BIOMARKERS

To quantify the repeated physical biomarkers, measurement techniques including pulse oximeters, immunostaining, and blood pressure cuffs were utilized (**Table 4**). Interestingly, both ABI and perfusion require a Doppler ultrasound to be quantified. Estimated glomerular filtration rate (eGFR) was generated from serum creatinine levels (a routine blood marker) and was therefore calculated from routine blood test results.

**Table 4:** Measurement techniques reported in included sources used to quantify repeated physical biomarker expression (ABI = ankle-brachial index; TcPO_2_ = transcutaneous oxygen pressure; SpO_2_ = saturation of peripheral oxygen; StO_2_ = skeletal muscle oxygen saturation; SPP = skin perfusion pressure; SBP = systolic blood pressure; DBP = diastolic blood pressure; eGFR = estimated glomerular filtration rate; H&E = hematoxylin and eosin; MTT = 3-[4,5-Dimethylthiazol-2-yl]-2,5-Diphenyltetrazolium Bromide).

Repeated Physical Biomarkers	Biomarker Measurement Techniques
Histology	Immunostaining of sectioned wound tissue samples using toluidine blue, Masson's trichrome stain, H&E stain, and primary antibody stains.
ABI	Vascular Doppler ultrasound.
TcPO_2_, SpO_2_, and StO_2_	Percutaneous oxygen partial pressure detector.
Perfusion (includes SPP)	Laser Doppler probe and blood pressure cuff.
Blood Pulse and Pressure Measures (includes SBP, DBP, toe pressure etc.)	Pulse oximeter.
eGFR	Calculated from routine blood test results.
Cell Viability	MTT (3-[4,5-Dimethylthiazol-2-yl]-2,5-Diphenyltetrazolium Bromide) assay, Trypan blue exclusion assay, and live/dead cell staining.

## DISCUSSION

### 1: KEY FINDINGS

This review identifies predictive, diagnostic, and/or indicative physical biomarkers of residual limb healing in adults with amputation, providing the foundation for the development of a standardized assessment scale for monitoring healing progression and prosthetic rehabilitation post-amputation.

Histological analysis, the most frequently reported biomarker, diagnoses cellular healing progression by quantifying key components such as collagen and keratinocyte presence which are crucial for all four wound healing phases. However, its need for wound tissue samples raises ethical and practical concerns, limiting its clinical application. Non-invasive hemodynamic and oxygenation biomarkers, such as transcutaneous oximetry, oxygen saturation measures, ABI, and skin perfusion pressure (SPP), provide valuable information regarding tissue oxygenation and vascular health, both of which predict and indicate healing outcomes. While eGFR serves as an indirect marker of kidney function that influences the healing process, it does not directly reflect the underlying mechanisms of healing. It identifies a comorbidity that may predict impaired healing, thus rendering it less useful for post-amputation assessments but valuable for pre-amputation risk assessment.

To enhance monitoring capabilities, there is a need for improved biomarker quantification techniques, such as the development of wearable sensors, as well as the utilization of multiple objective biomarkers to address the complex health considerations (comorbidities and heterogeneity) of individuals with amputation. There is a need for future research to determine biomarker threshold values for predicting, diagnosing, and indicating healing, ensuring their safe and effective application in the amputee population.

### 2: REPEATED PHYSICAL BIOMARKERS

#### 2.1: Physical Biomarkers

Histological analysis, utilizing techniques such as tissue sectioning, staining, and microscopic examination, provides cellular-level visual evidence of healing.^200^ Techniques like Masson's trichrome staining quantify collagen content,^201^ a crucial regulator in all wound healing phases.^202^ During the hemostasis phase, collagen promotes platelet activation and fibrin clot formation at the injury site. In the inflammatory phase, the activation of immune cells leads to the release of pro-inflammatory cytokines, which encourage fibroblast migration and collagen deposition.^202^ During proliferation, collagen degradation stimulates the production of growth factors and fibroblast proliferation, driving angiogenesis and re-epithelialization.^202^ Finally, during maturation, collagen composition alterations are essential for tissue remodeling and the tensile strength of healed skin. Bibi et al.^52^ utilized histological analysis to show that lapachol-treated mice with full-thickness wounds exhibited increased, organized collagen deposition and significant wound size reduction by days 8 and 10 post-wounding compared to controls (p < 0.001). Hematoxylin and eosin (H&E) staining serves to assess keratinocyte presence.^203^ Keratinocytes migrate into the wound to repair epidermal defects, and their proliferation, regulated by cytokines and growth factors, ensures complete wound coverage.^204^ Ferroni et al.^172^ employed H&E staining to assess diabetic foot ulcers (DFUs) treated with Therapeutic Magnetic Resonance (TMR(r)). DFUs treated with a non-functioning TMR(r) device exhibited a limited presence of fibroblasts, endothelial cells, keratinocytes, and collagen fibers (p < 0.001), which correlated with significantly longer healing times.^172^ The DFUs treated with an active TMR(r) device healed faster, averaging 44.8 ± 12.1 days versus 96.7 ± 23.5 days in the sham group (p < 0.05).^172^ Thus, histological analysis serves as a critical diagnostic tool for quantifying healing progression, particularly through the measurement of angiogenesis and collagen deposition at the wound site.

Estimated glomerular filtration rate (eGFR) is a quantitative measure derived from serum creatinine or cystatin C test results, serving as an indicator of kidney function by assessing the volume of blood filtered by the kidneys per minute.^205^ Its primary application is within observational studies concerning diabetic wounds, likely a consequence of the detrimental effects of diabetes on renal function.^206^ Chronic kidney disease (CKD) is characterized by a sustained reduction in eGFR to values below 60 mL/min/1.73 m² for a duration of three months or longer.^207^ The impact of CKD on wound healing is well-documented; findings from murine excisional wound models indicate that CKD-affected mice present altered blood chemistry and hematology profiles, reduced rates of re-epithelialization and granulation tissue deposition, and differential expression of genes associated with wound healing, including vascular endothelial growth factor, interleukin-1 beta, endothelial nitric oxide synthase, and inducible nitric oxide synthase.^208^ These changes are accompanied by significant reductions in cellular proliferation and angiogenesis, alongside heightened inflammatory responses when compared to control groups.^208^ Therefore, eGFR serves as an indicator of a comorbidity predictive of non-healing, making it less useful for post-amputation assessments but valuable for pre-amputation evaluations to identify patients at higher risk of impaired healing.

Cell viability is used only in bench research studies employing scratch assays, where healing is assessed by observing the migration of cells across a created “scratch” in the assay. In such studies, it is necessary to ensure the health of the cells to validate that the observed migration (or lack thereof) is a result of healing mechanisms, rather than poor cell culture conditions. Cell viability tests confirm this by quantifying the number of live/dead cells and/or the metabolic activity of the cells. Kasowanjete et al.^74^ for example, used Trypan blue stain to determine the number of viable cells in a cellular wound model investigating the impact of photobiomodulation at 660 nm on in vitro diabetic wound healing. Dead cells take up the dye due to permeable cell membranes, whereas the impermeable membranes of viable cells prevent them from taking up the dye. Cell viability is therefore diagnostic of cell health, and indicative of healing, but offers little clinical applicability to the amputee population. Instead, it is limited to use in preclinical research to evaluate the efficacy of novel therapeutic compounds designed to promote healing, or better understand the cellular level mechanisms that control healing in residual limb tissue.

Transcutaneous oxygen pressure (or transcutaneous oximetry [TcPO_2_]), peripheral oxygen saturation (or pulse oximetry [SpO_2_]), and skeletal muscle oxygen saturation (StO_2_) are non-invasive metabolic measures that provide insight into tissue oxygenation levels.^209^ Oxygen is critical for wound healing, influencing various stages of the healing process under both hypoxic and normoxic conditions.^209^ During the hemostasis phase, hypoxia plays a pivotal role in initiating the wound healing process by enhancing the activity of reactive oxygen species (ROS).^210^ In the inflammation phase, the elimination of bacteria occurs via phagocytosis, a process contingent upon high partial oxygen pressure.^211^ Vascular endothelial growth factor, a key growth factor in angiogenesis, is upregulated by hypoxia-inducible factor 1-alpha, which is activated by both hypoxia and ROS during the proliferation phase. In the maturation phase, which includes tissue remodeling, oxygen facilitates keratinocyte activity through ROS.^211^ Loo and Halliwel^212^ utilized a keratinocyte-fibroblast co-culture model of wound healing, to demonstrate hydrogen peroxide (H2O_2_), a common ROS, enhanced keratinocyte proliferation and accelerated the rate of epithelialization. Oxygen is evidently vital for facilitating cellular activity and tissue repair during healing, however techniques for assessing oxygen levels differ. For example, TcPO_2_ non-invasively quantifies local tissue perfusion via electrochemical sensors,^213^ with calf values exceeding 40 mmHg associated with a higher percentage of successful healing after below-the-knee amputation.^214^ Similarly, a retrospective study found a statistically significant relationship (p < 0.001) between lower TcPO_2_ values and prolonged wound healing duration in 84 patients with critical limb-threatening ischemia.^132^ Contrastingly, StO_2_ is assessed non-invasively through measurements of oxyhemoglobin and deoxyhemoglobin using near-infrared spectroscopy.^215^ Lee et al.^216^ demonstrated that skin wounded by pressure injuries exhibited a significantly higher median StO_2_ compared to healthy and scabbed skin. Thus, oxygenation measures function as predictive and indicative markers of healing post-amputation. They may also predict risk of further wounds to the residuum like deep tissue injury (DTI), caused by reduced oxygen levels resulting from vascular occlusions induced by loading during lower limb prosthetic use.^217^

The hemodynamic biomarkers, ankle-brachial index (ABI), perfusion, and blood pulse and pressure measures, indicate the vascular status surrounding a wound. Insufficient perfusion, characterized by poor macro-circulation, increases progressive hypoxia risk and diminishes nutrient and survival factors delivery necessary for tissue repair.^218^ This impairs processes such as angiogenesis, collagen deposition, and epithelialization, resulting in sustained inflammation. The angiogenesis phase of wound healing involves the formation of new blood vessels that supply nutrients, immune cells, and oxygen to the wound site.^219^ It is characterized by an initial period of rapid and excessive capillary growth that eventually regresses to a vascular density akin to that of normal skin.^219^ Therefore, hemodynamic measures are predictive and indicative of healing. For example, a systematic review indicated that an ABI value of less than 0.5 in patients with DFUs, calculated as the ratio of blood pressure in an ankle artery to that in an arm artery, was significantly associated with an increased incidence of major amputation.^220^ Skin perfusion pressure (SPP) of ≥ 40 mmHg and toe pressure of ≥ 30 mmHg (or ≥ 45 mmHg) were also linked to at least a 25% higher likelihood of healing. Similarly, a study of 81 diabetic patients concluded that normal ABI (0.90–1.30) correlated with successful healing (p < 0.05), while ABI (≤ 0.40) was associated with failed transmetatarsal amputation (p < 0.01).^183^

While valuable, hemodynamic measure interpretations vary. For instance, SPP evaluates vascularity by assessing the blood pressure required to restore microcirculatory or capillary flow after controlled occlusion, while ABI reflects the ratio of the ankle to arm blood pressure. The contrasting literature regarding each biomarker must be addressed. For example, calf TcPO_2_ values above 40 mmHg are associated with improved healing outcomes after below-the-knee amputation, while values below 20 mmHg correlate with poorer healing.^214^ However, a 2012 meta-analysis found insufficient evidence to establish an optimal TcPO_2_ threshold value for lower limb amputation clinical use.^25,214^ This review identifies the physical biomarkers commonly used in wound healing literature but highlights the need for further research to determine their threshold values, safety, and applicability in the amputee population.

#### 2.2: Quantification Techniques

The application of physical biomarkers in the proposed residual limb healing assessment scale is influenced by the methods used to quantify these biomarkers. Histological analysis offers the most detailed and diagnostic view of wound healing progression, but its quantification technique presents significant challenges. The requirement for wound tissue collection restricts histology's use primarily to bench research in animal models, as ethical concerns limit the use of human tissue samples.^221^ For example, in animal studies, such as that of Bibi et al.,^52^ tissue samples were collected at defined intervals (days 3, 7, and 10 post-wounding), allowing discrete snapshots of healing progression.

Conversely, hemodynamic and oxygenation measures were predominantly utilized in human participant studies, likely due to their non-invasive measurement techniques,^222^ ease of use, and incorporation into established clinical practice, such as ABI for peripheral arterial disease (PAD) assessment.^223^ Their non-invasive measurement techniques are however not immune to limitations. For example, ABI measurements require pressure to be applied to the limb, which can be painful in patients with ischemia or wounds,^222^ both of which are associated with amputation.^224,225^ TcPO_2_ measurements require the use of heated electrodes to enhance vasodilation,^214^ which may pose a risk of damaging sensitive post-operative residual limbs. Pulse oximetry is limited by poor peripheral perfusion, motion artefacts, and variations in skin pigmentation.^226^ These limitations introduce the need for improved biomarker quantification techniques specifically suited for residual limb monitoring, such as wearable wound healing sensors. For instance, Ochoa et al.^227^ are developing an integrated smart wound dressing capable of sensing and delivering oxygen to the wound.

Alternatively, employing a combination of biomarkers could provide a more comprehensive view of residual limb healing. Biomarkers are typically not exclusive to healing. For example, patients with lower extremity PAD, a common comorbidity among amputees,^228^ often present with TcPO_2_ calf values below 40 mmHg, while values above this threshold are generally associated with successful residual limb healing after below-the-knee amputation.^214^ To account for the comorbidities prevalent in the amputee population, multiple biomarkers should be utilized to provide a holistic view of residual limb health.

### 3: OVERALL SEARCH RESULTS AND STUDY CHARACTERISTICS

Most reviewed sources focused on diabetic wounds, a reflection of the global burden of diabetes, with an estimated 529 million individuals living with diabetes worldwide in 2021.^229^ DFUs are the most common complication of diabetes^230^ and a significant risk factor for amputation.^231,232^ For example, the Scottish Physiotherapy Amputee Research Group (SPARG) “Survey of the Lower Limb Amputee Population in Scotland 2019 Public Report” noted that over half (56%) of all lower limb amputees had the etiology of diabetes.^233^ Pre-amputation assessment is especially critical for patients with a greater number of comorbidities, such as diabetes, and suboptimal physiological factors known to predict wound complications.^12^ Diabetes can impair wound healing via hyperglycemia-induced vascular stiffening, microvascular dysfunction, and reduced oxygenation.^18^ Therefore, physical biomarkers may enhance pre-amputation assessments to improve post-amputation outcomes.

Age is another key factor affecting healing, with medians of the mean participant ages in included human studies ranging from 60.9 to 70.0 years, highlighting a predominance of older adults. Most non-healing wounds are a result of vascular disease,^234^ venous insufficiency,^235^ areas of high unrelieved pressure,^236^ diabetes,^237^ and disability;^238^ conditions that are increasingly prevalent as the population ages. For instance, Public Health England reports diabetes prevalence rising from 9.0% among individuals aged 45 to 54 years to 23.8% among those aged 75 years and over.^237^ Age-related factors, such as prolonged inflammation and increased production of reactive oxygen species during healing, can lead to chronic wounds.^239^ This aging effect is also reflected in the SPARG 2019 report, which found the median age at the time of lower limb amputation to be 67 years.^233^ As aging exacerbates healing complications and delays recovery, there is a critical need for objective measures of wound healing to accelerate prosthetic fitting and improve outcomes.

Gender also plays a significant role in predicting wound complications. An analysis of gender characteristics across human participant studies revealed that the median proportion of male participants ranged from 50% to 71%. Male gender is a risk factor for DFU development,^240^ poorer DFU healing,^241^ increased post-surgical infection rates,^242^ and higher in-hospital immortality rates after trauma.^243^ In the SPARG 2019 report, 71.5% of lower limb amputees were male,^233^ though studies also indicate that women may be less likely to successfully receive a lower limb prosthesis after amputation.^244^ These disparities highlight the need for gender-specific research^245^ and biomarkers not influenced by hormonal or gender-related factors.

Most studies did not investigate wound healing after amputation but focused on wounds in patient populations similar to those who undergo amputation, highlighting the lack of standardized approaches and understanding of the tissue changes that occur in residual limbs post-amputation. By extrapolating findings from wound healing studies in tissues and structures found in residual limbs, a foundational database of potential biomarkers can be established for use in residual limb healing. Notably, all studies on amputation included in this review examined lower limbs, which account for 4–5 times more amputations than upper limbs^246^ and face unique residual limb health requirements due to weight-bearing requirements during ambulation.

### 4: METHODOLOGICAL DISCUSSION

#### 4.1: Methodological Strengths

A broad exploration of the literature on biomarkers related to healing is provided in this review, allowing for the inclusion of diverse sources without strict criteria, unlike a systematic review which requires a focused research question. Instead, the findings can serve as a basis for subsequent systematic review, such as Johnson et al.'s review of IL-6 in wound healing,^247^ particularly if high-quality evidence on a specific biomarker emerges.

A notable strength of this review lies in its emphasis on the potential impact of biomarkers on the future of post-amputation healing and rehabilitation. By identifying physical biomarkers capable of diagnosing, identifying, or predicting healing, a starting point for further research into objective healing measures and quantification methodologies is provided. This moves us closer to a specific post-amputation residuum healing assessment scale, which may enable more timely healing interventions, enhancing non-healing prevention and treatment strategies.^248^

#### 4.2: Methodological Limitations

In this section limitations associated with specific study types, not explored in the Part 1 review,^23^ are discussed. Animal studies, despite genetic similarities to humans, often lack reliability due to biological differences and methodological issues,^249^ while mathematical models, although based on empirical data, can oversimplify the complexities of human biological processes.^250–252^ Consequently, biomarker behavior observed in both should be interpreted cautiously, serving as potential indicators rather than definitive predictors of human responses. All wound types affecting tissues relevant to the residuum were considered appropriate for inclusion in this review, however, future research needs to account for the differences between secondary intention healing wounds, like DFUs, and primary intention wounds, such as sutured surgical sites, when applying findings to clinical contexts.

The synthesis of data from diverse sources in scoping reviews risks oversimplification or loss of critical detail. Biomarkers that appeared repeatedly within and across different study types were prioritized for discussion in this review. However, this approach excludes biomarkers in only a single study or specific category. For example, Alfawaz et al.^184^ investigated tibial vessel run-off (VRO) and popliteal artery patency, reporting that higher VRO was associated with improved healing rates and shorter time to healing following below-knee amputation, and that preoperative popliteal patency was linked to higher postoperative ambulation rates. The study's solitary use of these biomarkers led to its exclusion from broader discussions. Yet, these findings suggest potential areas for future research given the statistically significant outcomes reported.^184^

The timing of biomarker quantification critically affects its diagnostic value; for example, hypoxia (low oxygen levels) is essential at the onset of healing, but prolonged low oxygen levels impeded healing.^253^ Future research should address the form of the biomarkers, the timing of their measurement, and the anatomical locations from which they are sampled to improve their relevance in clinic.

### 5: ETHICAL CONSIDERATIONS

In this review, evidence level was not utilized as an exclusion criterion, recognizing that recognizing that randomized controlled trials are the highest standard of evidence but are limited by high costs, restricted funding, and potential industry bias favoring positive results.^254^

Instead, the review focused on ensuring that all included studies clearly stated ethical approval and obtained informed consent from human participants, prioritizing ethical standards over rigid adherence to evidence hierarchies. Adulthood was defined as aged 18 years or older, acknowledging that global variations in defining adulthood exist (16 to 21 years),^255^ to prevent misinterpretation in international dissemination. Despite efforts to include grey literature in this review to broaden the scope and minimize bias,^256^ none of the sources identified met the inclusion criteria, primarily due to insufficient methodological transparency and the absence of explicit ethical approval.

## CONCLUSION

This scoping review aimed to identify predictive, diagnostic, and/or indicative physical biomarkers of healing within the tissues and structures of residual limbs in adults with amputation. The integration of various physical biomarkers into the assessment of healing in residual limbs post-amputation is paramount for optimizing patient outcomes. Histological analysis remains the gold standard diagnostic biomarker for evaluating cellular healing processes, particularly through the measurement of collagen and keratinocyte presence, but is limited by the ethical and practical challenges of using tissue samples from human subjects. Non-invasive indicative and predictive oxygenation and hemodynamic measures, such as transcutaneous oxygen pressure (TcPO_2_) and ankle-brachial index (ABI), provide valuable insights into tissue oxygenation and vascular health; however, further research is essential to establish specific threshold values and applicability within the amputee population. While the estimated glomerular filtration rate (eGFR) serves as an indirect marker of kidney function that influences the healing process, it does not directly reflect the underlying mechanisms of healing. Instead, it identifies comorbidities that may predict impaired healing, rendering it less useful for post-amputation assessments compared to other physical biomarkers. Nevertheless, eGFR remains advantageous for pre-amputation evaluations, particularly for identifying patients at heightened risk for impaired healing.

The findings underscore the global burden of diabetes, the role of age and gender disparities in wound healing, and the need for targeted research addressing these factors to improve post-amputation outcomes. Most included sources focused on wounds in populations common to those undergoing amputation, rather than directly examining post-amputation wound healing, highlighting a lack of understanding of the tissue changes that occur in residual limbs post-amputation. Developing a holistic residual limb specific healing assessment scale that integrates a diverse array of physical biomarkers (accounting for physiological factors and comorbidities known to influence healing) could substantially enhance our understanding of the healing process and inform the development of effective rehabilitation strategies for individuals undergoing amputation.

## DECLARATION OF CONFLICTING INTERESTS

The author has no conflicts of interest to declare.

## AUTHORS CONTRIBUTION

- **Hannelore Williams-Reid**: the primary author of the manuscript, undertook the scoping review and prepared the final manuscript as part of a 4-year PhD program.
- **Arjan Buis**: the primary PhD supervisor, assisted in developing the scoping review methodology and preparing the manuscript for publication.
- **Anton Johannesson**: the secondary PhD supervisor, assisted in developing the scoping review methodology and preparing the manuscript for publication.

All authors have read and approved the final version of the manuscript.

## SOURCES OF SUPPORT

The PhD project under which this scoping review/manuscript falls is funded by the UKRI EPSRC as part of the Centre of Doctoral Training (CDT) in Prosthetics and Orthotics (P&O) (studentship 2755854 “Wound management and early prosthetic rehabilitation“ within project EP/S02249X/1) and by Össur.

## Abbreviations & Acronyms:

**Abbreviations & Acronyms Definition**
ABI: Ankle-Brachial Index
C.G.: Control Groups
CKD: Chronic Kidney Disease
DBP: Diastolic Blood Pressure
DFUs: Diabetic Foot Ulcers
eGFR: Estimated Glomerular Filtration Rate
U.S. FDA: United States Food & Drug Administration
H&E: Haematoxylin and Eosin
H_2_O_2_: Hydrogen Peroxide
JBI: Joanna Briggs Institute
MTT Assay: 3-[4,5-Dimethylthiazol-2-yl]-2,5-Diphenyltetrazolium Bromide Assay
NHS: National Health Service
No.: Number
PAD: Peripheral Arterial Disease
PRISMA-ScR: Preferred Reporting Items for Systematic Review and Meta-Analyses for Scoping Reviews
ROS: Reactive Oxygen Species
SBP: Systolic Blood Pressure
SpO_2_: Saturation of Peripheral Oxygen
SPP: Skin Perfusion Pressure
StO_2_: Skeletal Muscle Oxygen Saturation
T.G.: Treatment Groups
TcPO_2_: Transcutaneous Oximetry
UK: United Kingdom
USA: United States of America
VRO: Vessel Run-Off
